# Assessing Heat Tolerance in Creeping Bentgrass Lines Based on Physiological Responses

**DOI:** 10.3390/plants12010041

**Published:** 2022-12-22

**Authors:** Qianqian Fan, David Jespersen

**Affiliations:** Department of Crop and Soil Sciences, University of Georgia, Griffin Campus, 1109 Experiment Street, Griffin, GA 30223, USA

**Keywords:** heat stress, creeping bentgrass, OJIP fluorescence

## Abstract

Heat stress is a major concern for the growth of cool-season creeping bentgrass (*Agrostis stolonifera* L.). Nonetheless, there is a lack in a clear and systematic understanding of thermotolerance mechanisms for this species. This study aimed to assess heat tolerance in experimental lines and cultivars to determine important physiological and biochemical traits responsible for improved tolerance, including the use of OJIP fluorescence. Ten creeping bentgrass lines were exposed to either control (20/15 °C day/night) or high temperature (38/33 °C day/night) conditions for 35 d via growth chambers at Griffin, GA. Principal component analysis and clustering analysis were performed to rank stress performance and divide lines into different groups according to their tolerance similarities, respectively. At the end of the trial, S11 729-10 and BTC032 were in the most thermotolerant group, followed by a group containing BTC011, AU Victory and Penncross. Crenshaw belonged to the most heat-sensitive group while S11 675-02 and Pure Eclipse were in the second most heat-sensitive group. The exceptional thermotolerance in S11 729-10 and BTC032 was associated with their abilities to maintain cell membrane stability and protein metabolism, plus minimize oxidative damages. Additionally, among various light-harvesting steps, energy trapping, dissipation and electron transport from Q_A_ to PQ were more heat-sensitive than electron transport from Q_A_ to final PSI acceptors. Along with the strong correlations between multiple OJIP parameters and other traits, it reveals that OJIP fluorescence could be a valuable tool for dissection of photosynthetic processes and identification of the critical steps responsible for photosynthetic declines, enabling a more targeted heat-stress screening. Our results indicated that variability in the level of heat tolerance and associated mechanisms in creeping bentgrass germplasm could be utilized to develop new cultivars with improved thermotolerance.

## 1. Introduction

As an important cool-season turfgrass native to Eurasia and North Africa, creeping bentgrass (*Agrostis stolonifera* L.) is widely used in high value turf areas across temperate regions of the globe, such as golf courses, due to its ability to tolerate low mowing heights and quick recovery from traffic and golf ball marks [1]. Although highly prized for its turf quality, creeping bentgrass has only low to moderate tolerance to high temperatures [2]. This makes heat stress a major concern in many transitional or sub-tropical areas such as the Southeastern China and the Southeastern United States where there are typically long hot summers combined with high temperatures, with damages being further exacerbated with more frequent and intense heat wave events as a function of climate change [3,4]. Many golf courses have been converted from creeping bentgrass to warm-season species, particularly bermudagrass (*Cynodon* sp.), due to a lack of heat tolerance in recent years [5].

High temperature can result in a number of physiological and biochemical injuries to plants, primarily including oxidative stress, photosynthesis inhibition and change in protein metabolism. Oxidative stress results from excess accumulation of reactive oxygen species (ROS) which are a group of free radicals, such as singlet oxygen (^1^O_2_)and hydroxyl radical (OH^−^) [6]. They can attack a range of essential cellular components, like proteins, carbohydrates, and lipids in particular to cause leakage of cellular contents, eventually leading to lipid peroxidation and decreased integrity of cell membranes [7]. Photosynthesis inhibition occurs when elevated temperature brings about damages to photosynthetic machinery, including chlorophyll breakdown and reduced photosystem II (PSII) activity [7]. In addition, change in protein metabolism is another common stress symptom. Heat stress generally causes decreased protein abundance, which has been stated in various cool-season turfgrasses including creeping bentgrass [7,8,9]. It impacts many important cellular activities including photosynthesis and oxidative stress. The D1 protein plays a key role in PSII repair. Damaged D1 protein undergoes proteolysis to be removed and then a newly synthesized D1 is assembled into PSII, thus recovering PSII activity [10]. One study on wheat (*Triticum aestivum*) suggested that faster turnover of D1 protein contributed to better PSII photochemical efficiency [11]. In the case of oxidative stress, new proteins are synthesized to defend against ROS, and oxidized proteins go on to degradation to be removed, which otherwise would accumulate, causing damage and cell death [12]. These negative effects ultimately result in declines in carbon stores, reduced growth, loss of green color, thinning of the turf canopy and eventual plant death. To this end, development of heat-tolerant creeping bentgrass cultivars is desperately needed.

A few defense pathways have been clarified to be common strategies responding to heat stress in turfgrasses, like enhanced ROS detoxification, greater maintenance of photosynthesis ability, or altered protein metabolism [7]. Nonetheless, it should be noted that the specific changes may differ between species, or even cultivars and genotypes, which plays a pivotal role in the wide divergence in thermotolerance [9,13,14]. Creeping bentgrass shows considerable intraspecific diversity among different lines for its tolerance to heat. Previous research has identified differences in germplasm for important stress-related traits and there is potential to develop new cultivars with improved ability to withstand high temperatures [15]. However, despite progress made, comparisons of specific mechanisms, and physiological and biochemical parameters of heat tolerance among creeping bentgrass germplasm are still limited and need to be explored further.

As noted previously, PSII inhibition is a typical heat-stress induced symptom. The chlorophyll fluorescence parameter, photochemical efficiency (TRo/ABS or Fv/Fm), reflects the quantum efficiency of energy trapping by PSII and has been widely used as a reliable and sensitive tool for stress detection in different plant species including creeping bentgrass [16,17,18,19]. A relatively new development in fluorescence methodology is OJIP fluorescence [20]. It monitors rise of fluorescence intensity to a maximum at various states [21]. The O state is dark-adapted state when all the reaction centers, quinone A (Q_A_), quinone B (Q_B_) and plastoquinone (PQ) are oxidized. Upon exposure to saturating light, electrons will migrate into the PQ pool via Q_A_ and Q_B_. When the majority of electrons have reduced Q_A_, the J state is reached (at ~2 ms). When Q_B_ molecules are also reduced, the I state is reached (at 30 ms). Lastly, the P state is reached when maximum fluorescence intensity is obtained with a concurrent peak reduction in PQ pool, regardless of exposure time. By studying the OJIP curve, multiple photosynthetic component processes unavailable through traditional fluorescence methodologies can be quantified, such as energy trapping by PSII photochemistry, energy dissipation in PSII antennae, as well as electron transport between PSII and photosystem I (PSI), thereby providing a deeper insight into the function of photosynthetic components that might impair plant performance due to unfavorable environmental conditions [22]. To date, the use of OJIP fluorescence in abiotic stress studies have been documented in quite a few species, like tomato [23], cotton [24,25] and soybean [26]. However, despite its wide-spread application in stress physiology, related reports in creeping bentgrass, to our knowledge, are non-existent.

A number of creeping bentgrass materials were previously screened for summer performance. Within this germplasm collection, several experimental lines were identified with exceptional level of thermotolerance and outperformed commercial cultivars currently available on the market [27]. Nevertheless, the specific physiological or biochemical responses involved in their enhanced tolerance to heat have not yet been clearly revealed. A more complete understanding of the mechanisms conferring improved thermotolerance is essential for the efficient development of elite cultivars. Hence, this project aimed to evaluate heat tolerance in various creeping bentgrass lines to confirm the exceptional performance under heat stress in these promising experimental lines, as compared to commercial cultivars that form a range of thermotolerance. A number of physiological and biochemical measurements, including OJIP fluorescence, were taken to explore the responses enabling superior lines to outperform others. Integration of multiple stress-related traits will shed further light on heat stress survival strategies in creeping bentgrass, and determine useful traits associated with stress tolerance which can be utilized to develop new cultivars with improved thermotolerance.

## 2. Results

Since significant effects of temperature, line, date, and their interactions were detected for most parameters (Table 1) and the focus is mainly on exploring variations among lines under stress, differences among lines were analyzed for a given day under individual treatment.

**Figure 1:**
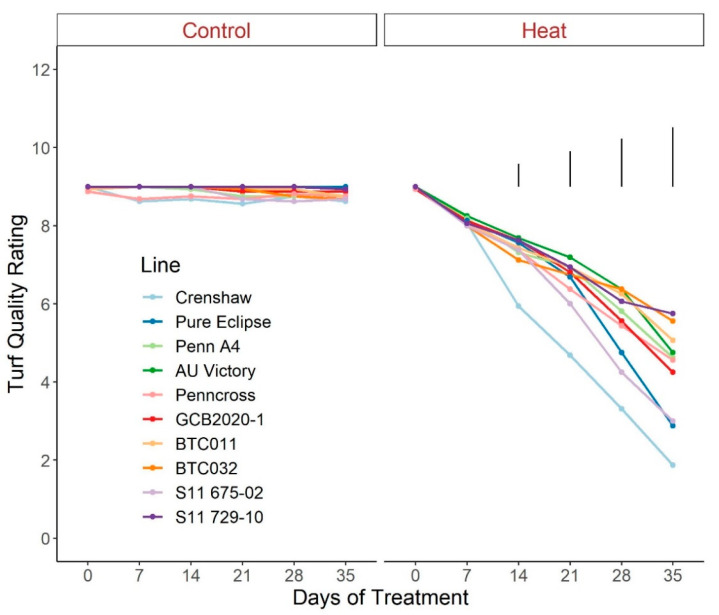
Change in visual turf quality rating for creeping bentgrass lines over time under control (20/15 °C day/night) and heat stress (38/33 °C day/night) conditions. Bars represent LSD values at *p* = 0.05 on days when significant differences among lines were found.

**Figure 2:**
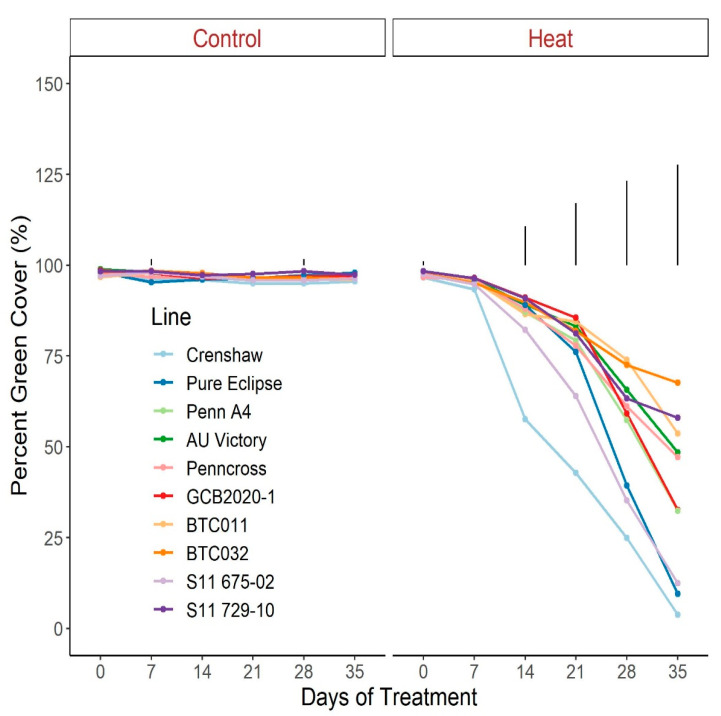
Change in percent green cover for creeping bentgrass lines over time under control (20/15 °C day/night) and heat stress (38/33 °C day/night) conditions. Bars represent LSD values at *p* = 0.05 on days when significant differences among lines were found.

For TQ, there were no significant differences for control plants over the duration of the trial, with all lines maintaining values greater than 8.5, representing a lack of stress (Figure 1; Table S1). Conversely, TQ scores declined throughout the trial for heat-stressed plants, to a greater extent in Crenshaw, Pure Eclipse as well as S11 675-02 than others, presenting variations in thermotolerance. These were in accordance with the significant effects of temperature and temperature × line interaction (Table 1). TQ scores were not significantly different among lines until heat progressed beyond 14 d, with differences being more pronounced over time. At the end of the trial, Crenshaw had the worst performance with an average score of 1.9 but not significantly differed from Pure Eclipse and S11 675-02. The two top performers, S11 729-10 and BTC032, had values of 5.8 and 5.6, respectively, without significant differences relative to BTC011, AU Victory, Penn A4 and Penncross. As with TQ, change in percent green cover followed a similar pattern (Figure 2; Table S2). All lines experienced significant drops in percent cover by 35 d of stress and the declines were greater in Crenshaw, Pure Eclipse and S11 675-02 than other lines. At 35 d, these three lines were also the poorest performers, whereas BTC032 was the top performer but was not significantly differed from S11 729-10, BTC011, AU Victory and Penncross.

**Figure 3:**
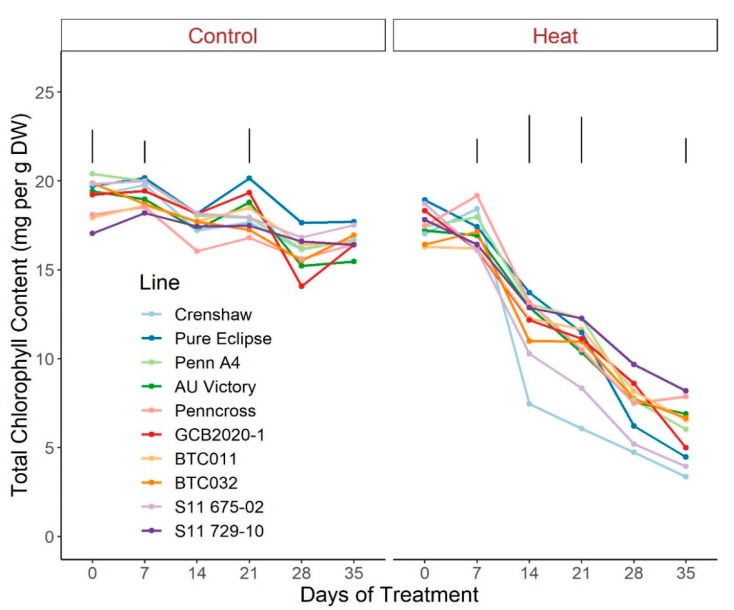
Change in total chlorophyll content for creeping bentgrass lines over time under control (20/15 °C day/night) and heat stress (38/33 °C day/night) conditions. Bars represent LSD values at *p* = 0.05 on days when significant differences among lines were found. DW, dry weight.

**Figure 4:**
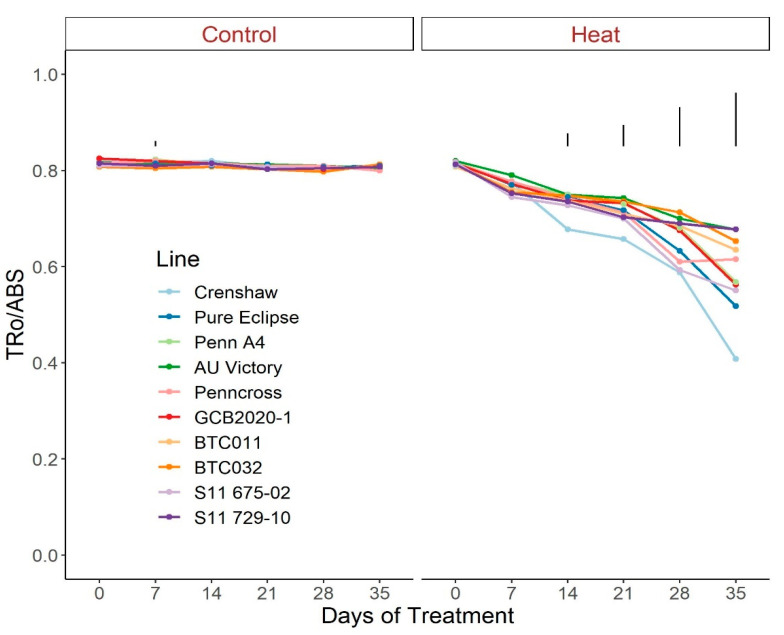
Change in Tro/ABS for creeping bentgrass lines over time under control (20/15 °C day/night) and heat stress (38/33 °C day/night) conditions. Bars represent LSD values at *p* = 0.05 on days when significant differences among lines were found.

**Figure 5:**
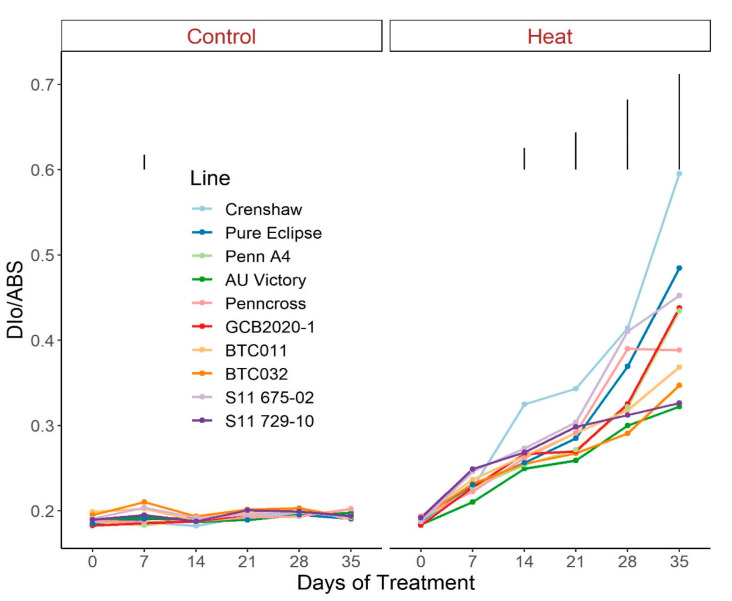
Change in Dio/ABS for creeping bentgrass lines over time under control (20/15 °C day/night) and heat stress (38/33 °C day/night) conditions. Bars represent LSD values at *p* = 0.05 on days when significant differences among lines were found.

**Figure 6:**
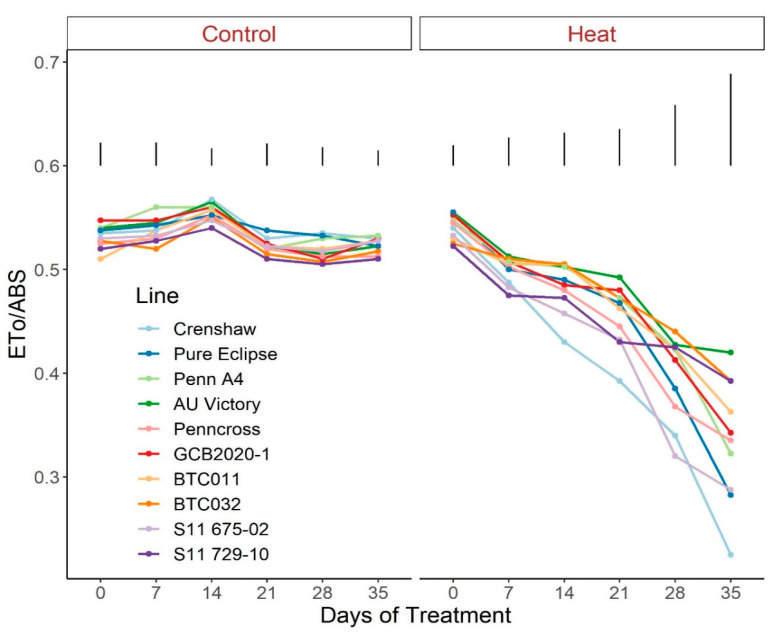
Change in Eto/ABS for creeping bentgrass lines over time under control (20/15 °C day/night) and heat stress (38/33 °C day/night) conditions. Bars represent LSD values at *p* = 0.05 on days when significant differences among lines were found.

Regarding photosynthetic attributes, consistent values of total chlorophyll content, TRo/ABS and DIo/ABS were maintained in most lines with little variation among lines on most sampling dates under control conditions (Figure 3, Figure 4 and Figure 5; Tables S3–S5). For ETo/ABS, no significant difference was found between 0 d and 35 d under control conditions despite variations over time, with differences among lines detected within sampling dates (Figure 6; Table S6). Intriguingly, Dio/ABS significantly rose while the other three parameters fell as a result of heat stress. At the end of week 5, the top statistical group contained S11 729-10, AU Victory, BTC032 and BTC011 for Tro/ABS as well as Dio/ABS measurements, S11 729-10, Penncross and AU Victory for chlorophyll content, and AU Victory, BTC032 and S11 729-10 for Eto/ABS. Crenshaw consistently presented the lowest values regarding Tro/ABS, total chlorophyll content and Eto/ABS but was not significantly different compared to Pure Eclipse for Tro/ABS, or to S11 675-02 and Pure Eclipse for chlorophyll levels or Eto/ABS at 35 d. Regarding Dio/ABS, the highest values were also found in Crenshaw at 35 d although it showed no significant difference compared to Pure Eclipse. As for Reo/ABS, only the main effect of date was significant.

**Figure 7:**
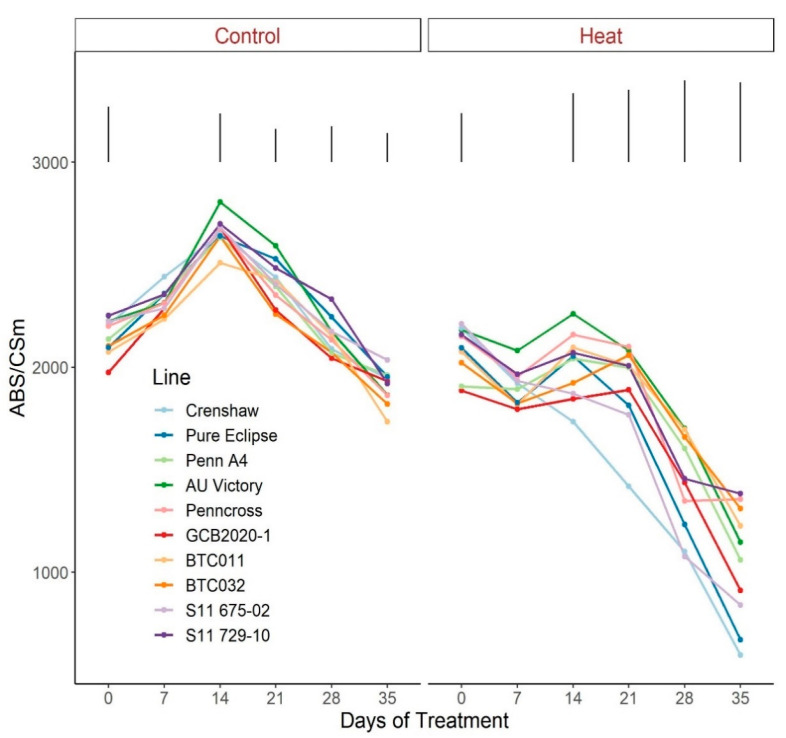
Change of ABS/CSm for creeping bentgrass lines over time under control (20/15 °C day/night) and heat stress (38/33 °C day/night) conditions. Bars represent LSD values at *p* = 0.05 on days when significant differences mong lines were found.

**Figure 8:**
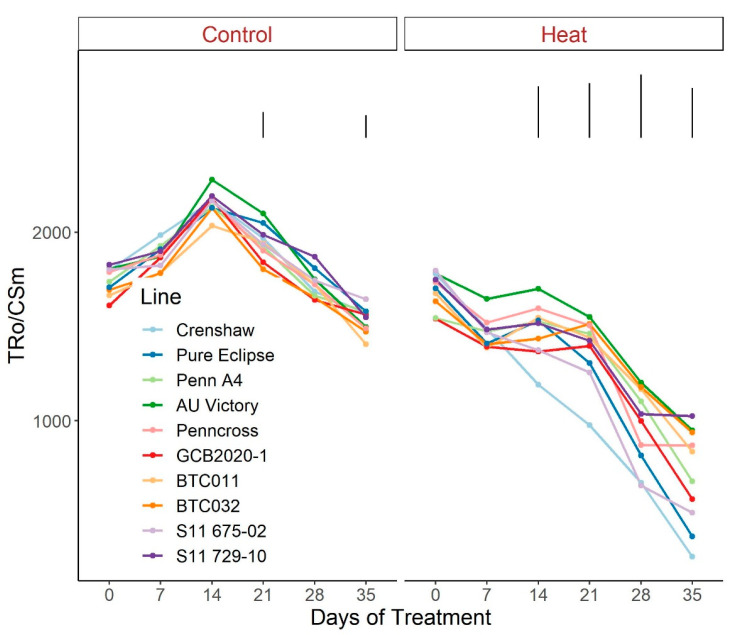
Change in Tro/CSm for creeping bentgrass lines over time under control (20/15 °C day/night) and heat stress (38/33 °C day/night) conditions. Bars represent LSD values at *p* = 0.05 on days when significant differences among lines were found.

**Figure 9:**
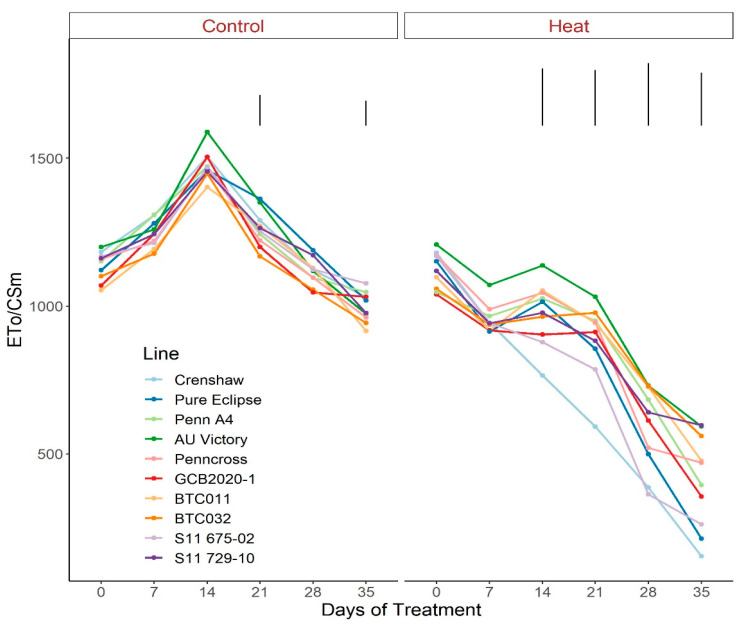
Change in Eto/CSm for creeping bentgrass lines over time under control (20/15 °C day/night) and heat stress (38/33 °C day/night) conditions. Bars represent LSD values at *p* = 0.05 on days when significant differences among lines were found.

As for the phenomenological energy fluxes involved in light-harvesting processes, significant differences were not found between 0 d and 35 d under control conditions for all parameters (ABS/CSm, Tro/CSm and Eto/CSm) although variations existed over time potentially as a consequence of chamber acclimation effects or natural genotypic variations, with differences detected within certain sampling dates (Figure 7, Figure 8 and Figure 9; Tables S7–S9). In contrast with control, heat stress caused significant reductions in every line for all three parameters, with pronounced separations being observed from 14 d onwards. At 35 d, Crenshaw, Pure Eclipse and S11 675-02 were the three poorest performers whose values were significantly lower than S11 729-10, Penncross and BTC032 in terms of ABS/CSm, and all the 10 emaining lines except for GCB2020-1 regarding Tro/CSm and Eto/CSm.

**Figure 10:**
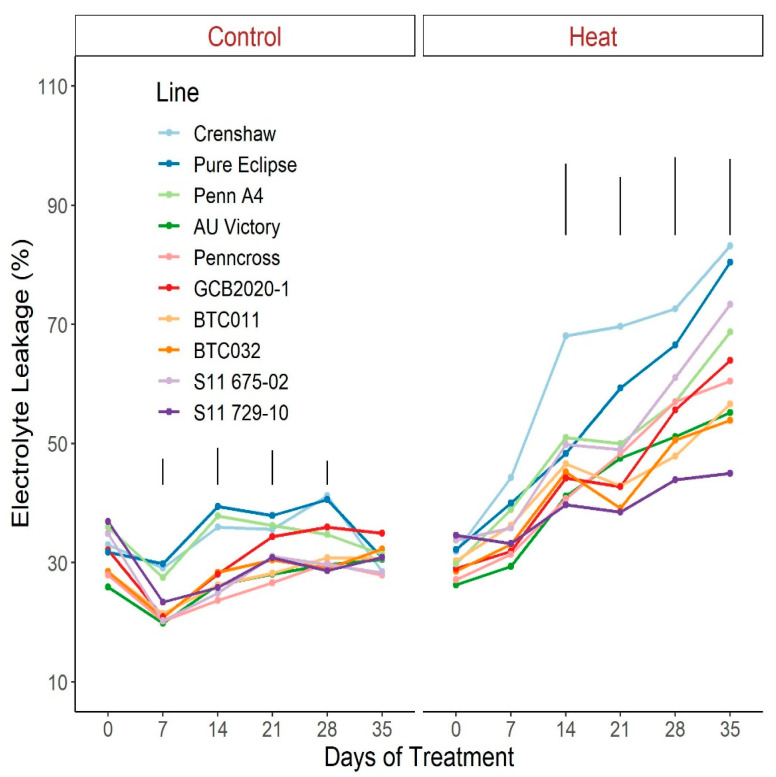
Change in electrolyte leakage for creeping bentgrass lines over time under control (20/15 °C day/night) and heat stress (38/33 °C day/night) conditions. Bars represent LSD values at *p* = 0.05 on days when significant differences among lines were found.

For EL under control conditions, no significant difference was seen between 0 d and 35 d despite some variance over time (Figure 10; Table S10). Contrastingly, values went up dramatically in response to heat stress in all lines with the exception of S11 729-10. Similar to most parameters mentioned above, variation among lines became apparent after two-weeks of stress with divergence increasing over time until 35 d. At 35 d of treatment, EL of S11 729-10 was the lowest but was not statistically different from BTC032, AU Victory, or BTC011, whereas Crenshaw had the highest value and was in the same statistical group as Pure Eclipse and S11 675-02.

**Figure 11:**
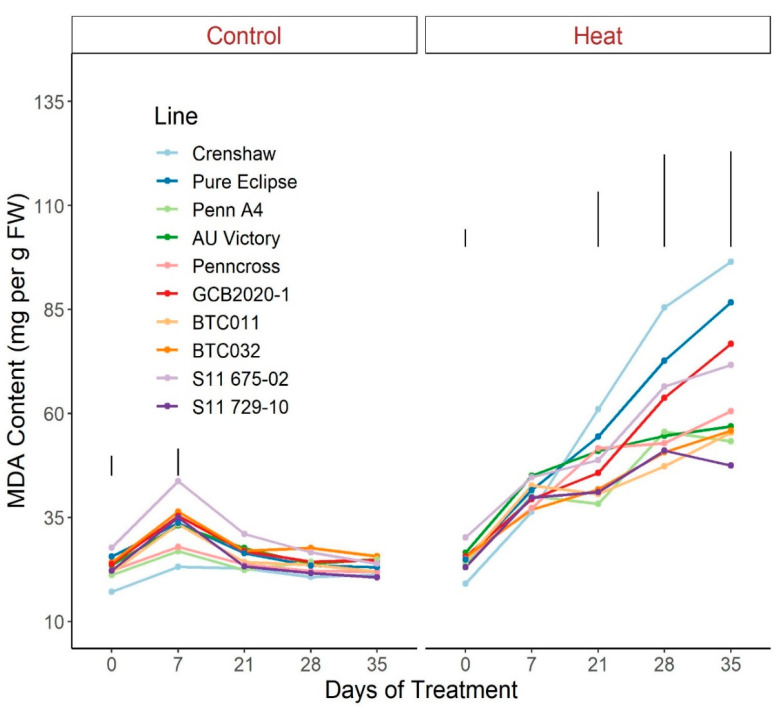
Change in MDA content for creeping bentgrass lines over time under control (20/15 °C day/night) and heat stress (38/33 °C day/night) conditions. Bars represent LSD values at *p* = 0.05 on days when significant differences among lines were found. FW, fresh weight.

For MDA content, plants under control conditions maintained mostly consistent values over time whereas significant rises were detected in all lines under heat stress, with the exception of S11 729-10 and AU Victory (Figure 11; Table S11). Pronounced variation among lines was found in response to stress at 21 d and continued to diverge through the end of the experiment. At 35 d, S11 729-10, as the top performer, presented a significantly lower MDA content than Crenshaw, Pure Eclipse, GCB2020-1 and S11 675-02.

**Figure 12:**
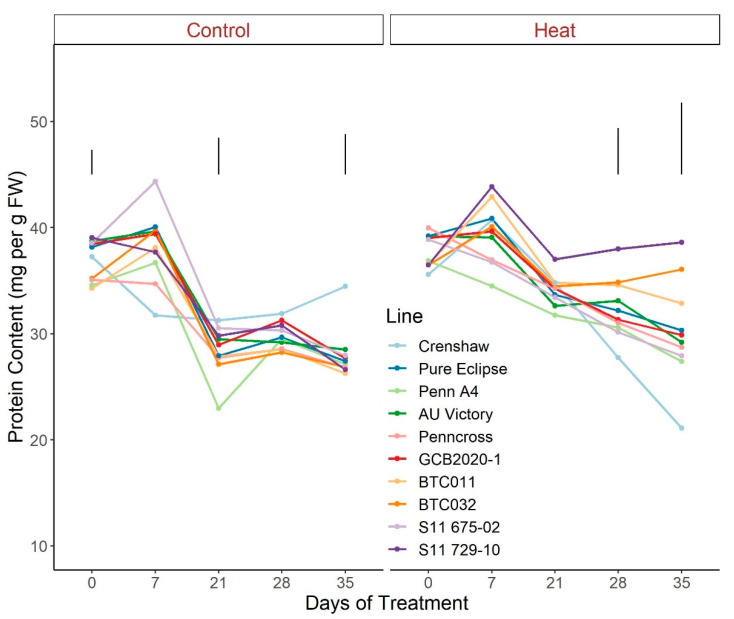
Change in total protein content for creeping bentgrass lines over time under control (20/15 °C day/night) and heat stress (38/33 °C day/night) conditions. Bars represent LSD values at *p* = 0.05 on days when significant differences among lines were found.

For protein abundance, plants followed similar patterns of change over time when exposed to control conditions, with greater values generally found at 0 d and 7 d potentially due to variations in fresh weight (Figure 12; Table S12). On the contrary, heat stress caused an obvious separation among lines with prominent differences detected over the last two weeks of stress. Specifically, at 35 d, S11 729-10 was the top performer, showing greater contents than the other lines with the exception of BTC032 and BTC011, whereas Crenshaw’s protein content was significantly lower than all others except Penn A4. Moreover, protein abundances presented no significant changes over the course of the five-week stress period for S11 729-10, BTC032 and BTC011 while the remaining lines all presented dramatic decreases.

For ESC, lines exposed to elevated temperature presented increases compared to those under control conditions, with an average of 81.0 and 37. 0 mg g^−1^ dry weight under heat stress and control conditions, respectively (data not shown). No significance differences existed for the line or line × temperature interaction effects. In this study ESC was not a useful parameter for separating heat tolerance among lines.

**Figure 13:**
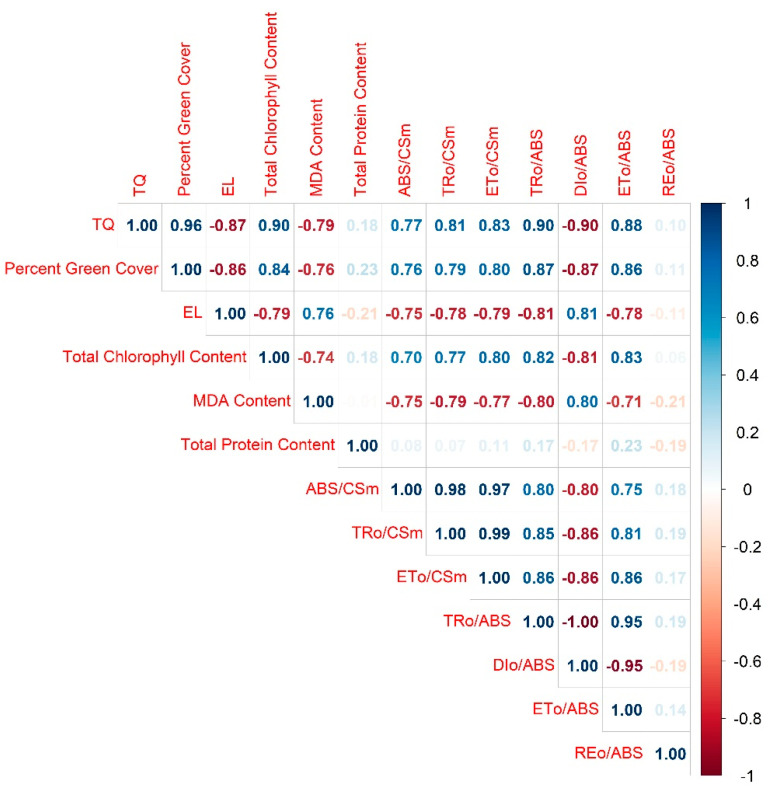
Correlation plot for different parameters of creeping bentgrass under control (20/15 °C day/night) and heat stress (38/33 °C day/night) conditions. Numbers indicate correlation coefficients. Color intensity is proportional to the correlation coefficients with blue indicating positive correlations and red representing negative correlations. Correlation coefficient values were left blank when not significant at *p* = 0.05.

Correlation analysis was conducted for all parameters except for ESC since there was limited data with no significant variation among lines. It revealed that all the parameters excluding protein content and REo/ABS were significantly and strongly correlated with each other, with the absolute values ranging from 0.70 to 0.99 (Figure 13). Conversely, the absolute values were not greater than 0.23 for the correlation coefficients between protein and other parameters, and not over 0.21 between REo/ABS and other parameters.

**Figure 14:**
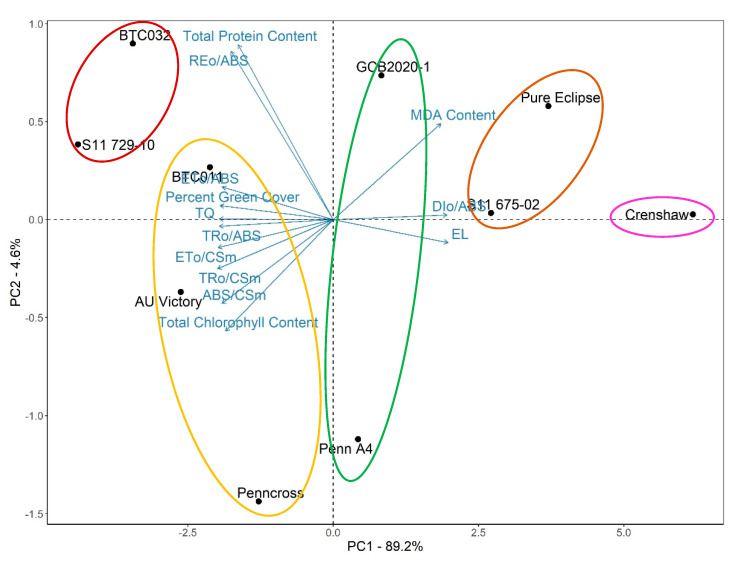
Principal component analysis for different parameters of creeping bentgrass at 35 d under heat stress (38/33 °C day/night) condition. Principal component 1 is represented on the *X* axis while principal component 2 is represented on the *Y* axis. Vectors indicate the direction and contribution of each parameter to the overall distribution of various lines. Circles of different colors indicate different clusters.

To take all the measurements into account to rank stress performance of lines, principal component analysis was conducted. Analysis determined the contribution of each component to the overall variation among lines due to differences after five weeks of heat stress, and also revealed to what extent different parameters contributed to stress tolerance (Figure 14). The first principal component (PC1) explained 89.2% of variance while the second principal component accounted for only 4.6% of variance. Except for total protein content and REo/ABS accounting for 5.7% and 6.6% of PC1, respectively, the contribution to PC1 made by the remaining traits ranged from 7.3 to 8.4%. Furthermore, clustering analysis was performed to divide lines into different groups according to their similarities (within-group variation is minimized). Together with results of principal component analysis, it revealed that S11 729-10 and BTC032 were in the most thermotolerant group. The second most thermotolerant group contained BTC011, AU Victory and Penncross, followed by the group containing Penn A4 as well as GCB2020-1. Crenshaw belonged to the most heat-sensitive group while S11 675-02 and Pure Eclipse were in the second most heat-sensitive group.

## 3. Materials and Methods

### 3.1. Growth and Treatment Conditions

A total of ten creeping bentgrass lines were used in this study, including five commercial cultivars (‘Crenshaw’, ‘Pure Eclipse’, ‘Penn A4′, ‘Penncross’ and ‘AU Victory’), and five experimental lines which have shown to perform well during summer in preliminary studies in Georgia, namely, ‘GCB2020-1′, ‘BTC011′ and ‘BTC032′ (Paul Raymer, unpublished work, 2020), plus ‘S11 675-02 ‘and ‘S11 729-10′ [27]. For each line, 6-cm-diameter plugs were established in plastic pots (10.5 cm long, 10.5 cm wide and 12.5 cm deep) filled with a mixture of 50% sand and 50% calcined clay (Turface; Profile Products LLC, Buffalo Grove, IL, USA) for ten weeks in greenhouse conditions [~23/~15 °C (light/dark period temperatures) and 70% relative humidity] before transferred to controlled environmental growth chambers (CG-72; Conviron, Winnipeg, MB, Canada). Plants were allowed one-week acclimation inside the growth chambers under conditions of 20/15 °C (day/night), 70% humidity and 14 h photoperiod with 600 µmol m^−2^ s^−1^ photosynthetically active radiation at the canopy level before the onset of different temperature treatments. Plants were maintained well-watered and fertilized weekly with a 24-8-16 (N-P-K) fertilizer (Scotts Miracle-Gro; Marysville, OH, USA) at the rate of 9.8 g N m^−2^ during establishment in the greenhouse as well as during the treatment period inside growth chambers. Applications of insecticide and fungicide were made as needed for disease control. Plants of each line were exposed to either heat stress (38/33 °C day/night) or control (20/15 °C day/night) conditions for 35 d after treatments began.

### 3.2. Measurements

#### 3.2.1. Physiological Measurements

Measurements consisted of assessments of whole-plant responses along with physiological and biochemical factors. Overall turf performance was estimated using a visual turf quality (TQ) rating on a scale of 1–9 and percent green cover via digital image analysis. Turf quality was determined according to color, density and uniformity with 1 representing totally dead grass, 9 standing for completely healthy grass with lush green color, and 6 being the minimum acceptable quality [28]. Digital image analysis was conducted through images taken with a digital camera (Canon G9X; Canon, Tokyo, Japan) using a lightbox to ensure a uniform lighting, which were processed using ImageJ v.1.46 to obtain values of percent green cover [29].

Total chlorophyll content and OJIP fluorescence were used to reflect the health status of photosynthetic machinery. Plants were dark adapted overnight (10 h) in growth chambers prior to performing OJIP measurements via a chlorophyll fluorometer set with a 3500 µmol actinic light intensity (OSP 5+; Opti-sciences, Hudson, NH, USA). This study focused on several energy flux and quantum efficiency parameters to better understand the light-harvesting processes, which included the energy flux absorbed by the antenna of PSII per cross section (ABS/CSm), the excitation energy flux trapped by open PSII reaction centers per cross section leading to the reduction of Q_A_ (TRo/CSm), the energy flux associated with electron transport from Q_A_ to PQ per cross section (ETo/CSm), quantum efficiency of energy trapping by PSII (TRo/ABS), quantum efficiency of energy dissipation in PSII antenna (DIo/ABS),quantum efficiency of electron transport Q_A_ to PQ (ETo/ABS), and quantum efficiency of electron transport Q_A_ to final PSI acceptors (REo/ABS) [22]. Four measurements were taken at the midpoint on fully expanded leaves for each replicate. To obtain values of total chlorophyll content, 0.1 g fresh leaves were incubated in 5 mL dimethyl sulfoxide for 7 days to allow for chlorophyll extraction. Then, the absorbance of solutions at 665 and 649 nm were read using spectrophotometer (Evolution 300 UV-visible spectrophotometer; Thermo Scientific, Madison, WI, USA) and converted to chlorophyll content according to previously derived equations on a dry weight basis [30].

Electrolyte leakage (EL) serves as an indicator of cell membrane stability. Around 0.1 g fresh leaves were placed in a tube containing 35 mL deionized water. After agitating tubes on a shaker for 16 h, initial conductivity was recorded through a conductivity meter (Radiometer, Copenhagen, Denmark). Next, the samples were autoclaved at 120 °C for 20 min, followed by incubation for another 16 h on a shaker, after which the final conductivity was read. EL then was calculated as the percentage of initial conductivity over total conductivity [31].

#### 3.2.2. Biochemical Measurements

Change in protein abundance was measured to represent change in protein metabolism, while malondialdehyde (MDA) content, a final product of lipid peroxidation, was quantified to indicate the extent of oxidative damage. Both analyses were performed through a microplate reader (Epoch 2 microplate reader, BioTek, Winooski, VT, USA). Approximately 50 mg fresh leaves were added into 1.1 mL 50 mM sodium phosphate buffer (pH 7.0 with 1 mM ethylenediaminetetraacetic acid). Supernatants were collected after homogenization and centrifugation at 15,000× *g*, 4 °C for 20 min. Then, total protein content was quantified at 595 nm with Bradford dye reagent and a bovine serum albumin standard [32]. For the quantification of MDA content, 0.25 mL supernatant was mixed and reacted with 0.5 mL reaction solution (20% *w*/*v* trichloroacetic acid and 0.5% *w*/*v* thiobarbituric acid) at 95 °C, followed by absorbance measurement at the wavelengths of 532 and 600 nm. MDA content was acquired by subtracting background absorbance at 600 nm from absorbance at 532 nm and then divided by an extinction coefficient of 155 mM^−1^ cm^−1^ [33].

The content of ethanol soluble carbohydrates (ESC) were determined based on the anthrone method [34]. Approximately 30 mg dry leaf tissues were homogenized in 5 mL of 95% (*v*/*v*) ethanol and centrifuged at 3500 rpm, 4 °C for 10 min. The pellet was washed with 5 mL of 70% (*v*/*v*) ethanol twice. Then, all soluble portions were pooled, vortexed and centrifuged again to remove debris. Next, 100 µL of the ethanolic extract was added into 3 mL of anthrone-sulfuric acid reagent (200 mg anthrone dissolved in 100 mL 72% (*v*/*v*) H_2_SO_4_). After incubation in boiling water for 10 min, the absorbance of the resultant reaction mixture was recorded at 630 nm, and compared against glucose standards in the range of 20–100 µg mL^−1^. All measurements were taken weekly except for contents of protein, MDA and ESC with the former two being measured every other week while the latter was analyzed once at the end of the trial.

### 3.3. Statistical Analysis

A completely randomized split-plot design was applied with temperature as the whole plot and line as the subplot, with each combination of temperature and line having four replications. During the trial, each temperature was repeated in four growth chambers. Inside each chamber, there were two pots for every line and the average of these two pots was used to represent an individual replicate.

Data were analyzed via ANOVA using a mixed model in JMP Pro 16.0.0 (SAS Institute Inc., Cary, NC, USA, 2021). Date, temperature, line, and their interactions were treated as fixed effects whereas experimental run and the whole plot were random effects. Before ANOVA, normal distribution of residuals and the homogeneity of variance were checked according to normal quantile-quantile plots and residuals versus fitted plots, respectively, to make sure data met ANOVA assumptions. Means were separated by Fisher’s protected least significant difference (LSD) at the 0.05 probability level. Correlation analysis and K-means clustering analysis were performed using corrplot and cluster packages, respectively, while principal component analysis was conducted through plotly and ggfortify packages in RStudio (R 3.6.0, Boston, MA, USA, 2019).

## 4. Discussion

Although elevated temperature caused damages to all plants over the course of the 35 d stress period, a wide range of thermotolerance was observed among lines as evidenced by the differences in their visual characteristics. Specifically, S11 729-10, BTC032, BTC011 and AU Victory were the four top performers, outperforming others by maintaining greater overall quality as measured by TQ and green cover. Conversely, heat-sensitive lines, such as Crenshaw, S11 675-02 and Pure Eclipse, consistently performed poorly in terms of these two measured parameters, while the remaining lines were intermediate in their performances. Moreover, superior visual characteristics in the more heat-tolerant lines were attributed to their improved physiological as well as biochemical responses. These included greater abilities to withstand injuries to photosynthetic machinery as reflected in chlorophyll content and OJIP fluorescence traits (TRo/ABS, DIo/ABS, ETo/ABS, ABS/CSm, TRo/CSm and ETo/CSm), maintain cell membrane stability as evaluated by EL, minimize oxidative damage as measured by MDA content, and reduce change in protein metabolism as indicated by total protein content.

Maintaining chlorophyll levels and chlorophyll fluorescence traits is critically important for cool-season grass survival during heat stress. The former contributes to the absorption of light energy for use in photosynthesis while the latter estimates the health of PSII reaction centers which are the most thermally labile component of the electron transport chain, with constraints in either of them impairing photosynthetic capacity [35,36]. Within this study, despite heat-induced declines in chlorophyll content, TRo/ABS, ETo/ABS, ABS/CSm, TRo/CSm and ETo/CSm and increases in DIo/ABS over time, more heat-tolerant lines S11 719-10, AU Victory, BTC032 and BTC011 generally better maintained these characteristics, revealing less damage to their photosynthetic systems, which is in accordance with previous research [16,37,38]. Chlorophyll loss is one major characteristic of leaf senescence induced by heat stress damage [39]. The lesser decline in chlorophyll content in heat-tolerant plants could be a consequence of slower chlorophyll degradation resulting from relatively lower gene expression levels of chlorophyll-degrading enzymes, like chlorophyllase, pheophytinase and chlorophyll-degrading peroxidases [40,41].

The light energy absorbed by photosynthetic pigments is either used in PSII photochemistry or dissipated through heat and fluorescence [42]. Energy absorption decreased as measured by ABS/CSm, potentially as a result of heat-induced chlorophyll reduction or damage to photosynthetic complexes. With reduced energy absorption, the resulting energy trapped by PSII reaction centers, as measured by TRo/CSm, would also be expected to go down, reducing the efficiency of trapping and ultimately causing declines in the light-harvesting abilities of the leaf [23]. As noted previously, electrons migrate from PSII to PSI via Q_A_ and PQ during light harvesting. When the electron flow to Q_A_ declined (TRo/CSm), there would generally be a concomitant decline in energy flux from Q_A_ to PQ too, as evaluated by ETo/CSm [23,43]. Likewise, when OJIP traits were expressed as energy fluxes per absorbed photo flux, declines in TRo/ABS and ETo/ABS were detected as well. A decrease in the quantum efficiency of light photochemical reactions in PSII (TRo/ABS) resulted in a rise of energy dissipation as heat and fluorescence, as evidenced by increases in DIo/ABS, highlighting that stress-induced damage required the leaves to dissipate excess excitation energy instead of utilizing it for photosynthetic processes [44]. Intriguingly, in contrast with the significances observed for TRo/ABS, DIo/ABS and ETo/ABS, neither temperature effects nor line effects were significant for the quantum efficiency of electron transport from Q_A_ to final PSI acceptors, as measured by REo/ABS, despite heat-induced numerical declines over time among all lines (data not shown). This suggested that during light-harvesting processes, energy trapping, energy dissipation and electron transport from Q_A_ to PQ were more sensitive to temperature rise than electron transport from Q_A_ to final PSI acceptors [23,45]. Identifying these critical steps responsible for photosynthetic inhibitions would allow for a more targeted screening and improvement of plants with enhanced heat tolerance.

Previous studies pointed out that heat stress impaired a range of OJIP fluorescence traits in croftonweed (*Ageratina adenophora*) and peony (*P. lactiflora*) [43,44]. However, more heat-tolerant populations or cultivars typically better maintained absorbed energy flux, energy flux trapped by PSII, electron transport from Q_A_ to PQ, as well as quantum efficiencies of energy trapping, dissipation, and electron transport from Q_A_ to PQ. Similarly, another study on tall fescue (*Festuca arundinacea* Schreb.), stated that at the end of heat treatments, the heat-tolerant “TF71” presented significantly higher values in absorbed energy flux, energy flux trapped by PSII, and quantum efficiencies of energy trapping and electron transport from Q_A_ to PQ than the heat-sensitive “TF133”, maintaining better photosynthetic capacity thus contributing to its enhanced adaptation to high temperature [38]. These are all in agreement with our findings. Additionally, the close associations among ABS/CSm, TRo/CSm, ETo/CSm, TRo/ABS, ETo/ABS and DIo/ABS were also supported by their significantly strong correlation coefficients among each other. Along with concurrent declines in ABS/CSm, TRo/CSm, ETo/CSm from 21 d onwards, and concurrent declines or increases in TRo/ABS, ETo/ABS and DIo/ABS from 7 d onwards, it could be inferred that injuries to photosynthetic components were wide spread in the chloroplast and these light-harvesting steps might be concomitantly damaged by heat stress. Furthermore, our study also detected strong correlations of physiological factors plus MDA content with all fluorescence traits except for REo/ABS. As a reliable parameter commonly used in heat stress screening, it was not surprising that TRo/ABS showed the strongest associations with other factors [14,17,46]. Nevertheless, it’s noteworthy that DIo/ABS had the same correlation coefficients as TRo/ABS did, indicating its potential as another rapid and reliable measurement for thermotolerance evaluation in turfgrasses. The second strongest correlations with physiological traits plus MDA content were observed for ETo/ABS, which were close to those for TRo/ABS and DIo/ABS. Stronger relationships between whole-plant seedling vigor and ETo/ABS than other OJIP traits were reported in cotton previously and the authors proposed that ETo/ABS could be used as a surrogate for more time-consuming seedling vigor measurement [24,25]. To our knowledge, this is the first time that the application of OJIP fluorescence in abiotic stress response has been reported in creeping bentgrass. It could serve as a valuable tool for actual dissection of photosynthetic processes and help understand which steps of light-harvesting electron transport were more sensitive to heat stress [43,44]. Such information would provide deeper insights into heat-induced photosynthetic declines, allowing for a more targeted screening and improvement of heat tolerance in plants.

Accumulated ROS triggered by heat stress can attack lipids, resulting in decreased membrane integrity and lipid peroxidation, so typically the EL value increases with a concomitant increase in MDA content during plants’ exposure to stress [16]. However, these responses could be specific at both species and cultivar levels with lower EL and MDA content representing improved thermotolerance [13,47]. This corroborates the results found in our study where, compared to heat-sensitive lines, lower values of EL and MDA content were detected in heat-tolerant lines at 35 d, particularly S11 729-10. S11 729-10 had relatively little increase in these two parameters during the entire period of stress, suggesting its superior ability to maintain cell membrane integrity and to minimize oxidative damage. Furthermore, although not measured in the current study, greater activities of antioxidant enzymes, for instance, superoxide dismutase, catalase, ascorbate peroxidase, and peroxidase, can result in lower MDA abundances in heat-tolerant lines by scavenging excess ROS to protect cells or macromolecules from severe oxidative damage [16,48]. This may also have contributed to better maintenance of chlorophyll content and fluorescence traits and lower EL, eventually leading to better overall quality. Strong correlations between MDA and many other physiological traits support that reduced oxidative damage may result in the maintenance of photosynthetic processes such as light harvesting, reduced cellular damage, and maintenance of overall turf quality.

Protein metabolism, a process controlled by the balance between protein synthesis and protein degradation, impacts many cellular activities such as the aforementioned photosynthesis and oxidative stress. Decrease in protein abundance is a typic stress-induced characteristic, which has been confirmed in a wide range of plant species besides creeping bentgrass, including but not limited to strawberry (*Fragaria* × ananassa cv. Camarosa) [49], tomato (*Lycopersicon esculentum* Mill.) and maize (*Zea mays* L.) [50]. In general, protein catabolism is accelerated to a greater degree compared to the biosynthesis process under unfavorable environmental conditions, taking major responsibility for reduced protein content in response to elevated temperature [51,52]. However, it was previously documented that heat-tolerant plants typically had lower declines in protein abundance [53,54]. This agrees with our findings among which, greater protein contents were seen in more heat-tolerant lines like S11 729-10 and BTC032 at the end of the experiment. The higher protein abundance in heat-tolerant plants could be a consequence of faster protein synthesis, slower protein degradation, or both. Proteins synthesized abundantly under heat are primarily heat shock proteins, functioning as chaperones by preventing other proteins from aggregation and refolding stress-damaged proteins [55], contributing to the maintenance of protein metabolism. Hence, a greater and earlier induction of heat shock proteins in the heat-tolerant plants could be one reason for their improved thermotolerance [53,56]. As an opposing process to protein synthesis, less protein catabolism or slower degradation may be due to reduced proteolysis activity carried out by the coordinated action of the ubiquitin-proteasome system and various proteases [57,58]. One previous study stated that less enhanced gene expression of cysteine protease and a slower rate of overall protein degradation were detected in heat-adapted *Agrostis scabra*, contributing to its higher protein thermostability, and thereby greater protein abundance compared creeping bentgrass [54]. Thus, in order to further understanding of protein regulation in response to heat stress in turfgrasses, more research is needed to understand proteolysis activity of both the ubiquitin-proteasome system and proteases.

As with the defense mechanisms discussed above, accumulation of sugars can be another important contributor to heat tolerance. It not only contributes to increased osmotic adjustment but also improves the integrity of cellular membranes, helping relieve plants from heat-induced damages, which has been documented in creeping bentgrass [59] and other plant species [60,61]. Moreover, ESC turned out to accumulate more in creeping bentgrass cultivars with better summer performance [14]. The authors proposed that elevated temperature resulted in rapid loss of water, causing dehydration, as manifested by the declines in leaf relative water content and osmotic potential, while accumulation of sugars could produce positive effects on water homeostasis, thus explaining the higher sugar contents along with better leaf water status and osmotic adjustment abilities found in the more heat-tolerant cultivars. These findings were not consistent with our research where no significance was shown among various lines in terms of ESC. The discrepancy between studies is likely due to differences in creeping bentgrass lines, environmental conditions such as stress duration and stress intensity, and measurement techniques.

## 5. Conclusions

In summary, a broad range of thermotolerance exists among creeping bentgrass lines At the end of the trial, the overall ranking for heat tolerance of lines was that S11 729-10 and BTC032 were in the most thermotolerant group while BTC011, AU Victory and Penncross were in the second most thermotolerant group; Crenshaw belonged to the most heat-sensitive group while S11 675-02 and Pure Eclipse were in the second most heat-sensitive group; The group containing Penn A4 and GCB2020-1 was intermediate in their tolerance ranking. The exceptional thermotolerance in S11 729-10 and BTC032 was mainly associated with their greater abilities to maintain integrity of cellular membranes, as well as protein metabolism, and the ability to minimize oxidative damages. In addition, among various light-harvesting steps, energy trapping, dissipation and electron transport from Q_A_ to PQ were more heat-sensitive than electron transport from Q_A_ to final PSI acceptors. Moreover, strong significant correlations were detected among multiple OJIP parameters and other stress-related factors. These suggest that OJIP fluorescence could be a valuable tool for dissection of photosynthetic processes and identification of the critical steps responsible for photosynthetic declines, enabling a more targeted screening and improvement of plants for enhanced heat tolerance. This is also the first time that the potential application of rapid OJIP assessments is addressed in creeping bentgrass. Additional research is needed to further reveal how heat-tolerant and heat-sensitive creeping bentgrass lines respond to high temperature from physiological, biochemical, and molecular mechanisms, particularly regarding proteolysis.

## Figures and Tables

**Table 1:** ANOVA results for heat stress trials of creeping bentgrass.

Parameter ^†^	*p* Value						
	Date (D)	Temperature (T)	Line (L)	D × T	D × L	L × T	D × T × L
TQ	<0.0001	0.0004	<0.0001	<0.0001	<0.0001	<0.0001	<0.0001
Percent green cover	<0.0001	0.0025	<0.0001	<0.0001	0.0002	<0.0001	<0.0001
EL	<0.0001	0.0064	<0.0001	<0.0001	0.0400	<0.0001	0.1994
Total chlorophyll content	<0.0001	0.0004	0.0044	<0.0001	0.4223	<0.0001	0.7669
MDA content	<0.0001	0.0004	0.0016	<0.0001	0.5540	0.0006	0.7585
Total protein content	<0.0001	0.0316	0.0002	<0.0001	0.7554	0.0001	0.0102
ABS/CSm	<0.0001	0.0008	0.0002	<0.0001	0.8551	0.0001	0.6623
TRo/CSm	<0.0001	0.0004	<0.0001	<0.0001	0.7140	<0.0001	0.4652
ETo/CSm	<0.0001	0.0005	0.0002	<0.0001	0.8837	<0.0001	0.7059
TRo/ABS	<0.0001	0.0010	<0.0001	<0.0001	<0.0001	<0.0001	<0.0001
DIo/ABS	<0.0001	0.001	<0.0001	<0.0001	<0.0001	<0.0001	<0.0001
ETo/ABS	<0.0001	0.0025	<0.0001	<0.0001	0.1282	<0.0001	0.0608
REo/ABS	<0.0001	0.0572	0.4620	0.0834	1.0000	0.1614	1.0000
ESC	\	0.0097	0.3803	\	\	0.9539	\

^†^ TQ, turf quality; EL, electrolyte leakage; MDA, malondialdehyde; ABS/CSm, the energy flux absorbed by the antenna of photosystem II (PSII) per cross section; TRo/CSm, the excitation energy flux trapped by open PSII reaction centers per cross section, leading to the reduction of quinone A (Q_A_); ETo/CSm, the energy flux associated with electron transport from Q_A_ to PQ per cross section; TRo/ABS, quantum efficiency of energy trapping by PSII; DIo/ABS, quantum efficiency of energy dissipation in PSII antenna; ETo/ABS, quantum efficiency of electron transport from Q_A_ to plastoquinone; REo/ABS, quantum efficiency of electron transport from Q_A_ to final photosystem I acceptors; ESC, ethanol soluble carbohydrate.

## Data Availability

Data is contained within the article and supplementary material.

## Supplementary Materials

The following supporting information can be downloaded at: https://www.mdpi.com/article/10.3390/plants12010041/s1, Table S1: Change in visual turf quality rating for creeping bentgrass lines over time under control (20/15 °C day/night) and heat stress (38/33 °C day/night) conditions; Table S2: Change in green cover (%) for creeping bentgrass lines over time under control (20/15 °C day/night) and heat stress (38/33 °C day/night) conditions; Table S3: Change in total chlorophyll content (mg per g DW) for creeping bentgrass lines over time under control (20/15 °C day/night) and heat stress (38/33 °C day/night) conditions; Table S4: Change in TRo/ABS for creeping bentgrass lines over time under control (20/15 °C day/night) and heat stress (38/33 °C day/night) conditions; Table S5: Change in ETo/ABS for creeping bentgrass lines over time under control (20/15 °C day/night) and heat stress (38/33 °C day/night) conditions; Table S6: Change in DIo/ABS for creeping bentgrass lines over time under control (20/15 °C day/night) and heat stress (38/33 °C day/night) conditions; Table S7: Change in ABS/CSm for creeping bentgrass lines over time under control (20/15 °C day/night) and heat stress (38/33 °C day/night) conditions; Table S8: Change in TRo/CSm for creeping bentgrass lines over time under control (20/15 °C day/night) and heat stress (38/33 °C day/night) conditions; Table S9: Change in ETo/CSm for creeping bentgrass lines over time under control (20/15 °C day/night) and heat stress (38/33 °C day/night) conditions; Table S10: Change in electrolyte leakage (%) for creeping bentgrass lines over time under control (20/15 °C day/night) and heat stress (38/33 °C day/night) conditions; Table S11: Change in MDA content (mg per g FW) for creeping bentgrass lines over time under control (20/15 °C day/night) and heat stress (38/33 °C day/night) conditions; Table S12: Change in protein content (mg per g FW) for creeping bentgrass lines over time under control (20/15 °C day/night) and heat stress (38/33 °C day/night) conditions.
