# Supplementary material for: Assessing Heat Tolerance in Creeping Bentgrass Lines Based on Physiological Responses
Source: Plants (Basel). 2022 Dec 22;12(1):41. doi: 10.3390/plants12010041 (PMC9824340; doi:10.3390/plants12010041)
Supplement: Supplementary file 1 [file plants-12-00041-s001.zip › plants-2102107-supplementary.pdf]

Table S1. Change in visual turf quality rating for creeping bentgrass lines over time under control (20/15°C day/night) and heat stress (38/33°C day/night) conditions

**Lines**	**Control**						**Heat**					
	**0**	**7**	**14**	**21**	**28**	**35**	**0**	**7**	**14**	**21**	**28**	**35**
Crenshaw	9.0	8.6	8.7	8.6	8.8	8.6	9.0	8.1	5.9 b	4.7 c	3.3 d	1.9 c
Pure Eclipse	9.0	9.0	9.0	9.0	9.0	9.0	9.0	8.1	7.6 a	6.7 ab	4.8 bc	2.9 bc
Penn A4	9.0	9.0	8.9	8.8	8.8	8.9	9.0	8.2	7.3 a	6.9 a	5.8 ab	4.6 a
AU Victory	9.0	9.0	9.0	9.0	8.9	8.9	9.0	8.3	7.7 a	7.2 a	6.4 a	4.8 a
Penncross	8.9	8.7	8.8	8.7	8.8	8.8	8.9	8.1	7.4 a	6.4 ab	5.4 abc	4.6 a
GCB2020-1	9.0	9.0	9.0	8.9	8.9	8.9	8.9	8.1	7.6 a	6.8 ab	5.6 ab	4.3 ab
BTC011	8.9	9.0	9.0	8.9	8.9	8.8	9.0	8.1	7.4 a	6.9 a	6.3 a	5.1 a
BTC032	9.0	9.0	9.0	8.9	8.8	8.7	9.0	8.0	7.1 a	6.8 ab	6.4 a	5.6 a
S11 675-02	9.0	9.0	9.0	8.7	8.6	8.7	9.0	8.0	7.4 a	6.0 b	4.3 cd	3.0 bc
S11 729-10	9.0	9.0	9.0	9.0	9.0	8.9	9.0	8.1	7.6 a	6.9 a	6.1 a	5.8 a
LSD	ns	ns	ns	ns	ns	ns	ns	ns	0.59	0.91	1.2	1.5

Note: values followed by a common lowercase letter within each column indicate no significant difference among various lines at $p = 0.05$; ns, not significant

Table S2. Change in green cover (%) for creeping bentgrass lines over time under control (20/15°C day/night) and heat stress (38/33°C day/night) conditions

**Lines**	**Control**						**Heat**					
	**0**	**7**	**14**	**21**	**28**	**35**	**0**	**7**	**14**	**21**	**28**	**35**
Crenshaw	98.0	96.3 bc	96.0	95.0	95.0 c	95.5	96.7 b	93.4	57.6 b	42.8 c	24.9 d	3.8 d
Pure Eclipse	98.1	95.4 c	96.1	96.5	97.3 ab	97.9	98.4 a	95.9	89.1 a	76.1 ab	39.3 bcd	9.6 cd
Penn A4	98.3	98.0 ab	96.6	96.1	96.4 bc	97.4	97.5 ab	95.7	87.0 a	79.3 ab	57.4 abc	32.4 bc
AU Victory	98.9	98.2 a	96.9	96.1	96.5 bc	96.9	98.1 a	96.2	89.3 a	83.2 a	65.8 a	48.5 ab
Penncross	97.9	96.8 abc	96.8	95.9	96.7 b	96.7	96.7 b	95.9	87.9 a	77.9 ab	61.1 ab	47.2 ab
GCB2020-1	98.6	97.9 ab	96.5	96.6	96.9 ab	97.0	97.8 a	96.5	91.1 a	85.5 a	59.2 ab	32.6 bc
BTC011	96.7	97.9 ab	97.2	96.6	96.2 bc	96.1	97.8 a	95.7	86.5 a	84.5 a	73.9 a	53.7 ab
BTC032	98.1	98.5 a	97.8	96.6	96.6 bc	96.3	97.7 ab	95.1	89.8 a	81.9 a	72.6 a	67.6 a
S11 675-02	97.1	98.1 a	96.8	95.9	95.9 bc	96.5	97.5 ab	94.8	82.2 a	64.0 b	35.2 cd	12.4 cd
S11 729-10	98.3	98.4 a	97.2	97.6	98.4 a	97.4	98.4 a	96.5	90.9 a	81.2 a	63.3 a	58.0 ab
LSD	ns	1.7	ns	ns	1.7	ns	1.0	ns	10.8	17.1	23.2	27.7

Note: values followed by a common lowercase letter within each column indicate no significant difference among various lines at $p = 0.05$; ns, not significant

Table S3. Change in total chlorophyll content (mg per g DW) for creeping bentgrass lines over time under control (20/15°C day/night) and heat stress (38/33°C day/night) conditions

Lines	Control						Heat					
	0	7	14	21	28	35	0	7	14	21	28	35
Crenshaw	19.6 abc	20.0 ab	17.2	17.7 bcd	16.2	16.7	17.0	18.8 ab	7.5 d	6.1 c	4.7 d	3.4 f
Pure Eclipse	19.7 abc	20.4 a	18.2	20.2 a	17.7	17.7	18.9	17.8 bcd	13.7 a	11.5 a	6.2 bcd	4.5 ef
Penn A4	20.4 a	20.3 a	18.1	17.9 bcd	16.2	16.6	17.5	18.3 abc	13.1 ab	12.3 a	7.7 abcd	6.0 cd
AU Victory	19.4 abc	19.2 abc	17.3	18.8 abc	15.2	15.5	17.2	17.3 cd	12.9 abc	10.4 ab	7.6 abcd	6.9 abc
Penncross	18.1 bcd	18.8 bc	16.1	16.8 d	15.6	16.5	17.5	19.5 a	13.2 ab	10.5 ab	7.5 abcd	7.9 ab
GCB2020-1	19.2 abc	19.7 abc	18.2	19.4 ab	14.1	16.4	18.3	16.5 d	12.2 abc	11.1 a	8.6 ab	5.0 de
BTC011	17.9 cd	18.9 bc	17.7	18.5 abcd	16.4	16.6	16.3	16.5 d	12.3 abc	11.7 a	8.2 abc	6.6 bc
BTC032	19.9 ab	19.0 bc	17.7	17.3 cd	15.5	17.0	16.4	17.5 bcd	11.0 bc	11.0 a	7.8 abc	6.7 bc
S11 675-02	19.8 ab	20.3 a	18.2	18.0 bcd	16.8	17.5	18.7	16.4 d	10.3 c	8.4 bc	5.2 cd	4.0 ef
S11 729-10	17.1 d	18.5 c	17.5	17.5 bcd	16.6	16.4	17.8	16.8 d	12.9 abc	12.3 a	9.7 a	8.2 a
LSD	1.9	1.3	ns	2.0	ns	ns	ns	1.4	2.7	2.6	ns	1.4

Note: values followed by a common lowercase letter within each column indicate no significant difference among various lines at $p = 0.05$; ns, not significant

Table S4. Change in TRo/ABS for creeping bentgrass lines over time under control (20/15°C day/night) and heat stress (38/33°C day/night) conditions

Lines	Control						Heat					
	0	7	14	21	28	35	0	7	14	21	28	35
Crenshaw	0.82	0.818 ab	0.82	0.81	0.81	0.81	0.81	0.78	0.68 b	0.658 b	0.588 c	0.41 d
Pure Eclipse	0.82	0.815 abc	0.81	0.81	0.81	0.81	0.82	0.77	0.75 a	0.718 a	0.633 abc	0.52 cd
Penn A4	0.81	0.823 a	0.81	0.81	0.81	0.81	0.82	0.78	0.75 a	0.730 a	0.680 ab	0.57 abc
AU Victory	0.82	0.813 abc	0.82	0.81	0.81	0.81	0.82	0.79	0.75 a	0.743 a	0.700 a	0.68 a
Penncross	0.82	0.82 ab	0.82	0.81	0.81	0.80	0.82	0.78	0.74 a	0.713 a	0.610 bc	0.62 abc
GCB2020-1	0.83	0.82 ab	0.82	0.81	0.80	0.81	0.82	0.77	0.74 a	0.733 a	0.675 ab	0.56 bc
BTC011	0.81	0.805 c	0.82	0.81	0.80	0.81	0.81	0.76	0.74 a	0.710 a	0.685 ab	0.64 ab
BTC032	0.81	0.805 c	0.81	0.80	0.80	0.81	0.82	0.76	0.75 a	0.735 a	0.713 a	0.65 ab
S11 675-02	0.81	0.810 bc	0.81	0.81	0.81	0.81	0.82	0.75	0.73 a	0.700 ab	0.593 c	0.55 bc
S11 729-10	0.82	0.810 bc	0.82	0.80	0.81	0.81	0.81	0.75	0.74 a	0.703 a	0.690 ab	0.68 a
LSD	ns	0.011	ns	ns	ns	ns	ns	ns	0.027	0.044	0.082	0.11

Note: values followed by a common lowercase letter within each column indicate no significant difference among various lines at $p = 0.05$; ns, not significant

Table S5. Change in ETo/ABS for creeping bentgrass lines over time under control (20/15°C day/night) and heat stress (38/33°C day/night) conditions

Lines	Control						Heat					
	0	7	14	21	28	35	0	7	14	21	28	35
Crenshaw	0.535 ab	0.538 abc	0.568 a	0.530 ab	0.535 a	0.530 ab	0.540 abc	0.488 abc	0.430 d	0.393 e	0.340 cd	0.225 d
Pure Eclipse	0.538 ab	0.543 abc	0.553 abcd	0.538 a	0.532 ab	0.523 abc	0.555 a	0.500 abc	0.490 ab	0.468 abc	0.385 abc	0.283 cd
Penn A4	0.540 ab	0.560 a	0.560 abc	0.520 ab	0.530 abc	0.533 a	0.548 ab	0.508 ab	0.503 ab	0.473 ab	0.423 ab	0.323 bc
AU Victory	0.540 ab	0.545 ab	0.565 ab	0.523 ab	0.515 bcd	0.523 abc	0.555 a	0.513 a	0.503 ab	0.493 a	0.428 a	0.420 a
Penncross	0.525 bc	0.530 bc	0.553 abcd	0.520 ab	0.513 cd	0.513 c	0.545 ab	0.503 ab	0.480 abc	0.445 bcd	0.368 bcd	0.335 abc
GCB2020-1	0.548 a	0.548 ab	0.560 abc	0.525 ab	0.510 d	0.530 ab	0.553 a	0.508 ab	0.485 abc	0.480 ab	0.413 ab	0.343 abc
BTC011	0.510 c	0.538 abc	0.558 abc	0.523 ab	0.520 abcd	0.528 ab	0.530 bc	0.505 ab	0.505 a	0.463 abcd	0.423 ab	0.363 abc
BTC032	0.528 abc	0.520 c	0.550 bcd	0.515 b	0.508 d	0.518 bc	0.525 c	0.510 a	0.505 a	0.473 ab	0.440 a	0.393 ab
S11 675-02	0.530 abc	0.533 bc	0.548 cd	0.523 ab	0.518 abcd	0.528 ab	0.533 bc	0.483 bc	0.458 cd	0.433 cd	0.320 d	0.288 cd
S11 729-10	0.520 bc	0.528 bc	0.540 d	0.510 b	0.505 d	0.510 c	0.523 c	0.475 c	0.473 bc	0.430 d	0.425 ab	0.393 ab
LSD	0.022	0.023	0.017	0.021	0.018	0.015	0.020	0.027	0.032	0.036	0.059	0.088

Note: values followed by a common lowercase letter within each column indicate no significant difference among various lines at $p = 0.05$; ns, not significant

Table S6. Change in DIo/ABS for creeping bentgrass lines over time under control (20/15°C day/night) and heat stress (38/33°C day/night) conditions

Lines	Control						Heat					
	0	7	14	21	28	35	0	7	14	21	28	35
Crenshaw	0.18	0.186 bcd	0.18	0.19	0.20	0.19	0.19	0.23	0.32 a	0.343 a	0.414 a	0.595 a
Pure Eclipse	0.19	0.191 bcd	0.19	0.19	0.20	0.19	0.19	0.23	0.26 b	0.285 bc	0.369 abc	0.484 ab
Penn A4	0.19	0.183 d	0.19	0.20	0.20	0.19	0.19	0.23	0.25 b	0.271 bc	0.322 bc	0.434 bcd
AU Victory	0.19	0.192 bcd	0.19	0.19	0.19	0.20	0.18	0.21	0.25 b	0.260 c	0.300 c	0.322 e
Penncross	0.19	0.187 bcd	0.19	0.19	0.19	0.20	0.19	0.22	0.26 b	0.291 bc	0.390 ab	0.388 bcde
GCB2020-1	0.18	0.185 cd	0.19	0.20	0.20	0.19	0.18	0.23	0.27 b	0.270 bc	0.325 bc	0.438 bcd
BTC011	0.20	0.202 abc	0.19	0.20	0.20	0.19	0.19	0.24	0.26 b	0.291 bc	0.318 bc	0.369 cde
BTC032	0.20	0.210 a	0.19	0.20	0.20	0.19	0.19	0.23	0.26 b	0.267 bc	0.291 c	0.347 cde
S11 675-02	0.19	0.204 ab	0.19	0.20	0.20	0.19	0.19	0.25	0.27 b	0.304 ab	0.410 a	0.452 bc
S11 729-10	0.19	0.195 abcd	0.19	0.20	0.20	0.19	0.19	0.25	0.27 b	0.298 bc	0.312 bc	0.326 de
LSD	ns	0.018	ns	ns	ns	ns	ns	ns	0.026	0.044	0.082	0.11

Note: values followed by a common lowercase letter within each column indicate no significant difference among various lines at $p = 0.05$; ns, not significant

Table S7. Change in ABS/CSm for creeping bentgrass lines over time under control (20/15°C day/night) and heat stress (38/33°C day/night) conditions

Lines	Control						Heat					
	**0**	**7**	**14**	**21**	**28**	**35**	**0**	**7**	**14**	**21**	**28**	**35**
Crenshaw	2205.4 ab	2440.4	2650.8 ab	2437.8 abcd	2088.9 bc	1944.0 ab	2191.4 a	1921.9	1733.2 c	1419.1 b	1100.3 c	595.5 c
Pure Eclipse	2095.5 ab	2359.0	2639.8 ab	2527.2 ab	2244.3 ab	1951.2 ab	2093.7 ab	1827.3	2053.5 abc	1812.2 a	1231.5 bc	670.0 c
Penn A4	2136.5 ab	2360.1	2632.9 ab	2393.8 bcde	2068.6 c	1961.1 ab	1906.2 b	1892.8	2041.7 abc	1998.3 a	1603.2 ab	1059.1 ab
AU Victory	2224.7 ab	2313.5	2804.6 a	2591.8 a	2173.5 abc	1864.6 bc	2182.3 a	2081.3	2260.7 a	2080.8 a	1701.0 a	1145.3 ab
Penncross	2200.4 ab	2312.5	2672.2 ab	2351.8 cde	2132.5 bc	1861.6 bc	2150.5 a	1954.3	2157.6 ab	2099.3 a	1346.6 abc	1356.2 a
GCB2020-1	1973.6 b	2286.3	2682.2 ab	2278.2 de	2042.7 c	1931.5 ab	1885.8 b	1794.9	1845.8 bc	1889.4 a	1436.4 abc	911.0 bc
BTC011	2072.9 ab	2234.5	2508.6 b	2424.5 bcd	2154.6 bc	1733.5 c	2075.7 ab	1816.8	2096.4 ab	2007.6 a	1696.2 a	1224.7 ab
BTC032	2102.6	2254.0	2637.6 ab	2258.0 e	2071.3 bc	1819.5 bc	2021.2 ab	1822.6	1922.8 bc	2057.7 a	1658.9 a	1310.3 a
S11 675-02	2222.8 ab	2286.7	2680.8 ab	2403.5 bcde	2174.5 abc	2035.3 a	2210.2 a	1933.3	1871.4 bc	1766.6 ab	1075.8 c	838.7 bc
S11 729-10	2251.5 a	2355.6	2698.1 ab	2484.1 abc	2331.0 a	1919.4 ab	2157.7 a	1966.0	2070.0 ab	2005.8 a	1455.6 abc	1382.0 a
LSD	271.1	ns	236.6	162.1	174.7	143.1	238.7	ns	335.4	352.4	398.6	388.7

Note: values followed by a common lowercase letter within each column indicate no significant difference among various lines at $p = 0.05$; ns, not significant

Table S8. Change in TRo/CSm for creeping bentgrass lines over time under control (20/15°C day/night) and heat stress (38/33°C day/night) conditions

Lines	Control						Heat					
	**0**	**7**	**14**	**21**	**28**	**35**	**0**	**7**	**14**	**21**	**28**	**35**
Crenshaw	1797.9	1984.5	2167.6	1967.1 abcd	1684.2	1570.6 ab	1779.3	1487.6	1191.4 c	976.0 c	672.1 cd	280.6 f
Pure Eclipse	1706.7	1909.7	2128.9	2049.2 ab	1808.9	1579.7 ab	1701.7	1410.0	1532.1 ab	1305.8 ab	815.4 bcd	386.2 ef
Penn A4	1736.8	1926.8	2134.5	1923.7 bcde	1663.5	1580.1 ab	1545.0	1473.4	1525.7 ab	1461.4 ab	1103.5 ab	679.2 bcd
AU Victory	1807.1	1870.6	2279.1	2100.0 a	1750.0	1496.8 bc	1781.7	1646.4	1700.0 a	1550.9 a	1203.1 a	949.2 a
Penncross	1788.9	1879.7	2168.0	1901.4 cde	1721.6	1488.8 bc	1734.6	1520.0	1596.4 ab	1505.4 ab	870.7 abcd	869.0 ab
GCB2020-1	1611.7	1866.5	2178.1	1840.2 de	1641.0	1563.6 ab	1541.2	1392.0	1367.2 bc	1396.1 ab	999.4 abc	585.0 cde
BTC011	1663.9	1785.0	2035.4	1946.0 bcd	1722.0	1406.5 c	1676.5	1386.7	1545.5 ab	1440.1 ab	1167.1 a	835.6 abc
BTC032	1695.0	1783.6	2129.0	1802.7 e	1654.0	1472.1 bc	1632.3	1405.8	1435.3 abc	1513.0 ab	1177.7 a	937.2 ab
S11 675-02	1803.6	1823.6	2168.2	1935.1 bcde	1743.6	1644.4 a	1796.8	1468.2	1374.6 bc	1256.3 bc	656.7 d	513.6 def
S11 729-10	1828.0	1890.0	2190.9	1987.2 abc	1870.7	1548.4 ab	1747.8	1484.0	1517.5 ab	1424.1 ab	1036.8 ab	1024.4 a
LSD	ns	ns	ns	137.2	ns	120.6	ns	ns	274.1	290.8	336.9	265.3

Note: values followed by a common lowercase letter within each column indicate no significant difference among various lines at $p = 0.05$; ns, not significant

Table S9. Change in ETo/CSm for creeping bentgrass lines over time under control (20/15°C day/night) and heat stress (38/33°C day/night) conditions

Lines	Control						Heat					
	0	7	14	21	28	35	0	7	14	21	28	35
Crenshaw	1182.8	1307.5	1506.3	1289.8 abc	1122.7	1030.8 abc	1180.1	943.4	765.2 c	592.5 c	386.1 c	153.7 f
Pure Eclipse	1121.9	1279.8	1460.1	1363.0 a	1188.5	1019.1 abcd	1151.5	914.4	1014.5 ab	855.4 ab	499.1 bc	213.4 ef
Penn A4	1151.4	1309.0	1472.4	1243.6 cd	1095.9	1047.2 ab	1047.0	966.0	1025.3 ab	950.1 ab	683.7 ab	395.1 bcd
AU Victory	1200.0	1258.9	1588.6	1350.8 ab	1119.1	973.9 bcde	1207.9	1070.9	1137.6 a	1031.6 a	730.5 a	592.8 a
Penncross	1158.8	1220.0	1468.2	1221.9 cd	1096.0	961.3 cde	1172.1	989.5	1045.1 ab	944.8 ab	520.5 abc	470.4 abc
GCB2020-1	1069.7	1244.5	1504.3	1199.3 cd	1045.8	1031.1 abc	1039.5	918.1	904.0 bc	912.6 ab	612.3 ab	355.5 cde
BTC011	1053.8	1193.6	1403.1	1271.3 abcd	1129.4	916.0 e	1097.5	917.6	1053.1 ab	942.6 ab	726.2 a	476.9 abc
BTC032	1101.1	1177.3	1448.1	1167.9 d	1055.7	942.5 de	1058.6	937.5	964.1 ab	977.4 a	729.1 a	560.4 ab
S11 675-02	1168.3	1213.9	1469.5	1253.6 bcd	1124.7	1076.9 a	1169.2	943.0	877.4 bc	786.0 b	363.5 c	262.1 def
S11 729-10	1162.0	1243.9	1455.1	1263.9 abcd	1172.2	976.5 bcde	1118.3	941.8	977.1 ab	882.7 ab	640.8 ab	596.9 a
LSD	ns	ns	ns	103.6	ns	84.7	ns	ns	193.3	188.0	211.0	180.0

Note: values followed by a common lowercase letter within each column indicate no significant difference among various lines at $p = 0.05$; ns, not significant

Table S10. Change in electrolyte leakage (%) for creeping bentgrass lines over time under control (20/15°C day/night) and heat stress (38/33°C day/night) conditions

Lines	Control						Heat					
	0	7	14	21	28	35	0	7	14	21	28	35
Crenshaw	33.0	29.1 a	35.9 a	35.6 abc	41.2 a	30.5	31.9	44.3	68.0 a	69.7 a	72.6 a	83.2 a
Pure Eclipse	31.7	29.8 a	39.4 a	37.9 a	40.6 a	35.8	32.2	40.0	48.3 b	59.3 b	66.5 ab	80.5 ab
Penn A4	36.0	27.5 ab	37.8 a	36.3 ab	34.7 bc	33.4	29.8	38.9	51.0 b	50.0 bc	57.0 bcd	68.7 bcd
AU Victory	25.9	19.8 c	26.3 b	28.0 d	29.7 d	30.5	26.3	29.4	41.1 b	47.5 cde	51.1 cd	55.2 ef
Penncross	27.9	20.2 c	23.7 b	26.6 d	29.9 d	27.9	27.1	31.4	40.7 b	48.2 cd	57.0 bcd	60.5 de
GCB2020-1	32.1	20.9 c	28.1 b	34.4 abc	35.9 b	34.9	29.0	31.9	44.2 b	42.7 cde	55.6 bcd	63.9 cde
BTC011	28.4	21.4 c	26.3 b	28.2 d	30.8 cd	30.7	30.3	36.2	46.6 b	42.9 cde	47.9 d	56.7 def
BTC032	28.5	20.8 c	28.3 b	30.4 cd	29.0 d	32.2	28.6	33.1	45.2 b	39.2 de	50.5 cd	53.9 ef
S11 675-02	33.6	20.3 c	24.9 b	31.0 bcd	29.7 d	28.3	33.8	35.8	49.8 b	49.0 c	61.0 abc	73.3 abc
S11 729-10	36.9	23.3 bc	25.8 b	30.8 bcd	28.7 d	30.9	34.6	33.2	39.7 b	38.5 e	43.9 d	45.0 f
LSD	ns	4.5	6.2	5.8	4.0	ns	ns	ns	12.0	9.7	13.1	12.8

Note: values followed by a common lowercase letter within each column indicate no significant difference among various lines at $p = 0.05$; ns, not significant

Table S11. Change in MDA content (mg per g FW) for creeping bentgrass lines over time under control (20/15°C day/night) and heat stress (38/33°C day/night) conditions

Lines	Control					Heat				
	0	7	21	28	35	0	7	21	28	35
Crenshaw	17.1 c	23.2 e	22.8	20.7	21.2	19.1 c	36.4	61.0 a	85.4 a	96.5 a
Pure Eclipse	25.6 ab	33.7 bcd	26.4	23.4	23.0	24.9 b	41.6	54.5 ab	72.7 ab	86.7 ab
Penn A4	21.1 bc	26.9 de	22.3	24.4	24.9	23.8 b	40.2	38.3 d	55.6 bc	53.3 de
AU Victory	23.5 ab	33.1 bcd	27.7	23.9	24.9	26.5 ab	45.0	51.0 abcd	54.7 bc	56.9 cde
Penncross	22.2 b	28.0 cde	23.7	22.1	22.0	25.0 b	37.3	51.7 abc	52.8 bc	60.5 cde
GCB2020-1	23.8 ab	35.4 b	26.9	24.4	24.7	25.5 b	39.4	45.7 bcd	63.7 abc	76.8 abc
BTC011	21.7 bc	33.4 bcd	24.3	23.6	22.0	24.6 b	42.7	40.6 cd	47.3 c	55.4 cde
BTC032	24.2 ab	36.4 b	27.0	27.6	25.7	25.6 b	36.9	41.8 bcd	50.7 bc	55.8 cde
S11 675-02	27.8 a	43.7 a	31.0	26.6	23.9	30.2 a	44.7	48.8 abcd	66.5 abc	71.6 bcd
S11 729-10	22.2 b	35.0 bc	23.2	21.7	20.6	23.1 bc	39.8	41.1 cd	51.1 bc	47.5 e
LSD	4.8	6.5	ns	ns	ns	4.2	ns	13.3	22.2	23.0

Note: values followed by a common lowercase letter within each column indicate no significant difference among various lines at $p = 0.05$; ns, not significant

Table S12. Change in protein content (mg per g FW) for creeping bentgrass lines over time under control (20/15°C day/night) and heat stress (38/33°C day/night) conditions

Lines	Control					Heat				
	0	7	21	28	35	0	7	21	28	35
Crenshaw	37.3 ab	31.8	31.3 a	31.9	34.5 a	35.6	40.7	34.8	27.8 d	21.1 d
Pure Eclipse	38.2 a	40.1	27.9 ab	29.7	27.4 b	39.2	40.9	33.7	32.2 bc	30.3 bc
Penn A4	34.6 c	36.7	23.0 c	29.5	27.1 b	36.9	34.5	31.7	30.6 bcd	27.4 cd
AU Victory	38.7 a	39.7	29.5 ab	29.2	28.5 b	39.2	39.1	32.6	33.1 bc	29.2 c
Penncross	35.1 bc	34.7	27.8 b	28.6	26.8 b	40.0	37.0	34.3	31.0 bcd	28.7 c
GCB2020-1	38.5 a	39.4	29.0 ab	31.3	27.7 b	39.0	39.7	34.3	31.3 bcd	29.9 bc
BTC011	34.3 c	38.1	27.9 ab	28.5	26.3 b	36.6	42.9	34.8	34.6 ab	32.9 abc
BTC032	35.2 bc	39.8	27.1 b	28.3	26.9 b	36.5	40.1	34.5	34.9 ab	36.1 ab
S11 675-02	38.6 a	44.4	30.5 ab	30.3	28.0 b	38.9	36.8	33.4	30.2 cd	27.9 c
S11 729-10	39.1 a	37.7	29.8 ab	30.8	26.6 b	36.5	43.9	37.0	38.0 a	38.6 a
LSD	2.3	ns	3.5	ns	3.8	ns	ns	ns	4.4	6.8

Note: values followed by a common lowercase letter within each column indicate no significant difference among various lines at $p = 0.05$; ns, not significant
